# What is driving the resurgence and persistence of vaccine-targeted pneumococcal serotypes?—Serotype 19F as the paradigm

**DOI:** 10.1371/journal.ppat.1014065

**Published:** 2026-03-19

**Authors:** Deus Thindwa, Paloma M. Carcamo, Ron Dagan, Daniel M. Weinberger

**Affiliations:** 1 Department of Epidemiology of Microbial Diseases and the Public Health Modeling Unit, Yale University, New Haven, Connecticut, United States of America; 2 The Shraga Segal Department of Microbiology, Immunology and Genetics, Faculty of Health Sciences, Ben-Gurion University of the Negev, Beer-Sheva, Israel; University of Mississippi Medical Center, UNITED STATES OF AMERICA

## Abstract

Over the past 25 years, pneumococcal conjugate vaccines (PCVs) have markedly reduced both pneumococcal disease and nasopharyngeal carriage caused by vaccine serotypes among the more than 100 known pneumococcal serotypes. In the United States, the transition from the original 7-valent formulation (PCV7) to the 13-valent vaccine (PCV13) occurred approximately a decade after the initial introduction of PCVs, whereas several other countries implemented higher-valency formulations over shorter time intervals. More recently, next-generation PCVs targeting 15 (PCV15) or 20 (PCV20) serotypes have been introduced into pediatric immunization programmes, along with a novel 10-valent PCV designed for use in low- and middle-income countries. These vaccines are also now available for use in older adults, including a 21-valent formulation (V116) that targets a distinct set of serotypes. Since the introduction of PCVs, the composition of pneumococcal serotypes responsible for severe disease has changed substantially. In many settings, several vaccine-targeted serotypes have been nearly eliminated as causes of disease; however, the incidence of disease caused by certain non-vaccine serotypes has increased. In some settings, like the United States, serotypes that were initially suppressed following PCV7 introduction have subsequently re-emerged and again constitute major causes of disease, despite the continued use of PCVs that include those serotypes. The mechanisms underlying this resurgence of vaccine-targeted serotypes remain poorly understood. Elucidating the processes that drive these patterns is critical for assessing whether additional serotypes may re-emerge in the future and for identifying strategies to mitigate such increases. Here, we outline several hypotheses regarding potential mechanisms contributing to serotype resurgence and discuss how vaccine characteristics and serotype-specific traits may shape future pneumococcal population dynamics. We also identify key data gaps and priority research questions that must be addressed to improve understanding of serotype resurgence.

## Introduction

Pneumococcal conjugate vaccines (PCVs) were first introduced for widespread use in infants 25 years ago in the United States (US) [1]. The first generation of PCVs targeted seven of 100+ serotypes and, within just a few years, these seven serotypes were nearly eliminated as causes of disease in the US [2]. Because PCVs also prevent colonization of the upper respiratory tract, transmission of these serotypes was also reduced [3]. As a result, the decline in rates of disease caused by PCV-targeted serotypes was seen both in young, vaccinated children and in older children and adults who were not vaccinated [3]. This indirect protection greatly contributed to the public health benefits of PCV programs. In fact, the vast majority of prevented cases was in unvaccinated older individuals [4]. In the subsequent years, many other countries have introduced PCVs [5,6], with notable declines in PCV-targeted serotypes among vaccinated children. The degree and speed at which PCV-targeted serotypes declined among unvaccinated age groups differed between populations. Indirect effects tended to be slower and less dramatic in certain locations with higher transmission and vaccination schedules that did not include a booster [7–9].

The original PCV7 was subsequently supplanted by PCV10 (GSK) and PCV13 (Pfizer). In the US, the switch from PCV7 to PCV13 occurred 10 years after the original introduction. In many other countries, there was a shorter period between when PCV7 was introduced and when they switched to the new PCV. More recently, next-generation PCVs, which include 15 serotypes (Merck) or 20 serotypes (Pfizer), have been introduced in pediatric immunization schedules [10,11]. Another new PCV includes 10 serotypes that are of greatest concern in lower- and middle-income countries (PCV10, Serum Institute of India) [12].

At the same time, PCVs are also available for use in older adults and those with certain high-risk conditions. Options for vaccinating adults include using the same PCV15 or PCV20 as in the pediatric population, as well as a 21-valent vaccine (Merck) that targets a different set of serotypes and is used exclusively in adults [13,14]. These new vaccines have the potential to further address the remaining burden of disease caused by pneumococcus in children and adults.

In the years since PCVs were first introduced, there have been many dynamic changes to the population of serotypes causing severe disease. While some vaccine-targeted serotypes were nearly eliminated as causes of disease in many populations, the frequency of disease caused by some serotypes not targeted by vaccines increased in many populations [3,15]. In some places, certain serotypes that were initially suppressed by PCV7 subsequently resurged and have again become major causes of disease, despite continued use of PCVs that target those serotypes in the population [16–18]. This phenomenon of resurgence of vaccine-targeted serotypes is not well understood. Understanding the mechanism driving these patterns is critical to determine whether additional serotypes might resurge in the future and how such increases could be prevented.

In this commentary, we first summarize what is known about post-vaccine serotype dynamics of pneumococcus, including both vaccine-targeted serotypes and non-vaccine serotypes. We then present several hypotheses for mechanisms that could be driving these resurgence patterns and how characteristics of the different PCVs could influence future patterns of serotype dynamics.

## Section 1: Serotype competition, serotype resurgence, and differences between PCVs

*Memory B cells, antibodies, and capsule characteristics determine protection against colonization and disease.* PCVs induce the production of antibodies that target the polysaccharides of specific serotypes. These antibodies reduce the risk of acquiring pneumococcus in the upper respiratory tract (colonization) and the risk of progressing from colonization to disease [19,20]. PCVs, unlike pneumococcal polysaccharide vaccines, also generate serotype-specific memory B cells, providing longer-term protection [21,22]. For some serotypes, especially 19F, weaker memory responses are generated, resulting in a shorter period in which sufficient protective antibodies are produced. Some serotypes, including 19F, also require higher antibody concentrations to achieve protection against colonization and invasive pneumococcal disease (IPD) [23]. This is likely related to characteristics of the bacteria, such as the thickness or composition of the bacterial capsule [24]. Notably, prevention of carriage is typically associated with a higher concentration of efficacious antibodies compared to prevention of disease, thus vaccines that generate lower antibody concentrations may not effectively prevent colonization yet still provide protection against invasive disease [23,25,26].

*Consequences of ecological competition between serotypes*. Pneumococcus colonizes the nasopharynx of young children. The bacteria compete to occupy the nasopharynx, and some serotypes are more competitive than others [27,28]. This competition has direct implications for serotype patterns after PCVs are introduced. PCV-induced serotype-specific immunity prevents colonization by the targeted serotypes, thus reducing competition in the nasopharynx and making space for serotypes not targeted by the vaccine. As a result, declines in rates of colonization with serotypes targeted by PCVs have been quickly offset by increases in rates of colonization with non-targeted serotypes (serotype replacement) [15,29]. Therefore, in most locations, the overall frequency of pneumococcal colonization in children did not change, or changed only minimally, but the mix of serotypes did. Fortunately, as a group, the serotypes that increased in frequency among colonized children had usually a lower tendency to cause severe disease [30,31]. Therefore, despite these increases in rates of colonization by non-vaccine serotypes, overall rates of invasive disease still declined compared to the pre-PCV period. The degree to which increases in the frequency of non-vaccine serotypes offset declines in the frequency of PCV7-targeted serotypes differed by population and could partially be explained by the pre-vaccine frequency of different serotypes [32].

Beyond serotype replacement, competition between serotypes might have had an under-appreciated effect on reducing the prevalence of vaccine-targeted serotypes. Mathematical models suggest that the observed reductions in colonization by vaccine-targeted serotypes resulted from a combination of immunological protection against acquiring vaccine-targeted serotypes as well as protection resulting from competition from non-vaccine serotypes [33,34].

Thus, further suppression of the acquisition of vaccine-targeted serotypes were likely due to both non-vaccine serotypes that have the ability to outcompete vaccine serotypes and non-vaccine serotypes that needed a little competitive advantage by eliminating vaccine serotypes, as is evident from non-vaccine serotypes that emerged after PCV introduction.

**Fig 1:**
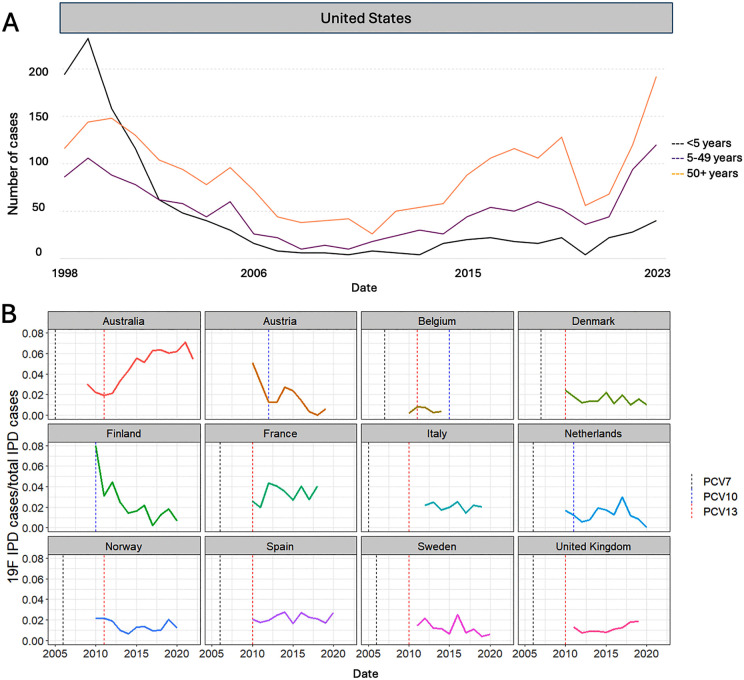
Trends in invasive pneumococcal disease (IPD) cases due to 19F across several countries. **(A)** The decline in the number of reported 19F IPD cases after introducing PCV7 in 2000 (black dotted line) in the United States and subsequent rebound of 19F IPD cases since switching to PCV13 in 2010 (red dotted line). **(B)** Time series of 19F IPD cases in different countries in all ages where the first dotted line represents the introduction (and a second dotted line represents a switch in some countries) of PCVs according to each country’s PCV schedules. These data are from the CDC (https://data.cdc.gov/Public-Health-Surveillance/1998-2023-Serotype-Data-for-Invasive-Pneumococcal-/qvzb-qs6p/about_data) and the European Centers for Disease Prevention and Control (https://atlas.ecdc.europa.eu/public/index.aspx).

*Reduction and resurgence of serotype 19F.* In many settings, the incidence of serotype 19F fell sharply after the introduction of PCVs and has remained at low frequency. However, after 19F was initially reduced by more than 50% as a cause of IPD in the US from 2004-2012 due to PCV7 [35], the frequency of adult IPD cases caused by 19F in 2019 (*n* = 64) and 2023 (*n* = 96) is now as high or higher than it was before the introduction of PCV7 in 1998–1999 (*n* = 65) (Fig 1A) [36]. The increase in IPD caused by 19F started in the US around 2012 and has accelerated in recent years [35]. The start of this increase coincided with the switchover from PCV7 to PCV13 in the pediatric population in 2010 and the implementation of a sequential schedule for PCV13/PPSV23 in high-risk adults in 2012 and all adults ≥65 years in 2014 [37]. The increased rates of IPD due to 19F have been predominant among unvaccinated older children and adults. However, serotype 19F has remained rare among children <5 years of age, the group that has been most recently immunized with PCVs. The increases in the incidence of 19F have also occurred despite the use of PCV13 in older adults [37], or variations in vaccine schedules (e.g., three-primary doses + booster in the US or two-primary doses + booster in South Africa).

In multiple locations, serotype 19F was reported to resurge or persist during the post-PCV13 period. Similar to the US, data from Australia demonstrates a strong increase in IPD caused by 19F, starting around the same time as the increase in the US [38,39]. However, the resurgence in serotype 19F has not been universal. Among the larger European countries that report data to the European Center for Disease Control and Prevention [40], 19F remained at a low level through 2019, comprising <4% of IPD cases (Fig 1B). Even within the US, some regions have reported larger increases than others [35]. After England adopted the reduced 1 + 1 PCV schedule in January 2020, an increase in 19F IPD cases was observed, alongside serotypes 3, 19A, and 4, consistent with clonal expansion based on whole-genome sequencing results [41].

Colonization studies provided some of the earliest indications of the issues with protection against 19F. In Alaska, a notable increase in colonization with 19F was reported during 2012–2013 compared with 2008–2009, predominantly among healthy older children and adults, and largely confined to one of the three communities studied [16]. Subsequent studies involving children in Saint Louis, Missouri (USA), and adults in New Haven, Connecticut (US) also reported high levels of colonization with serotype 19F during the post-PCV13 period, despite high uptake of PCVs in both populations [17,42]. In South Africa and Malawi, 19F has remained an important colonizing serotype [43,44], and has persisted as the most important serotype targeted by PCV13 that continues to cause disease in South Africa [43,45]. In The Gambia, serotype 19F colonization has remained the most prevalent among both younger and older children, even after 14 years of routine PCV13 use in the pediatric population [46]. In a separate colonization study from England, serotypes 19F, 3, and 19A were among the earliest acquired in fully vaccinated toddlers, suggesting limited duration of memory B cell-mediated immunity and a need for high antibody concentrations for effective protection against these serotypes [47].

While the most widespread example, serotype 19F is not the only PCV-targeted serotype that has resurged. Serotype 4, also included in both PCV7 and PCV13, has shown a marked increase in incidence in recent years. However, its epidemiological pattern has been distinct from that of 19F. The resurgence of serotype 4 has primarily resulted from localized clusters of disease. In the US, this is predominant in the Western states and among high-risk populations such as individuals experiencing homelessness and Indigenous communities [48–50]. In Finland, serotype 4 outbreaks have been reported among shipyard workers [51,52]. In Belgium, the absolute number of IPD cases caused by several PCV serotypes (particularly 4, 12F, and 14) has surged dramatically since 2020 [53]. Data from the Public Health Agency of Canada also showed increasing trend in serotypes 4, 9V, 19F, and 12F from 2018-2022 [54].

*Variability in PCV coverage, PCV immune response, and PCV schedules*. The different PCVs that have been used over the past 25+ years have partial overlap in which serotypes are included. There are also some differences in serotype coverage, with newer PCVs either covering more serotypes (PCV13, PCV15, PCV20) [1,10,11], or targeting the epidemiology in specific regions or risk groups (PCV10/Pneumosil for use in low/middle income countries; PCV21 for use in adults) [12,13]. The PCVs differ in the strength of the immune response they elicit, as measured by antibody responses following the infant and/or toddler doses of the vaccine [23,25]. Typically, PCVs that target more serotypes generate lower immune responses against each serotype, but this is not always the case, and for serotype 19F, PCV13 and PCV10 were reported to generate a more robust immune response compared with PCV7, especially after the booster [22].

In addition to differences in which serotypes are included in PCVs, there are also important differences between countries in how these vaccines are used. PCV7 was originally introduced with three primary doses, administered to infants, and a booster dose administered around the first birthday (3 + 1 schedule) [1]. Many countries opted to introduce the vaccine instead with three primary doses and no booster dose (3 + 0 schedule) or with two primary doses and a booster dose (2 + 1 schedule) [6,7,55]. Recently, some countries have moved to a single primary dose and a booster dose (1 + 1 schedule) [56]. Moreover, several other middle-income countries in the process of transitioning from Gavi support are exploring the feasibility of implementing fractional dosing strategies to mitigate the costs associated with PCV [57]. These different schedules differ in terms of the immune response generated. For instance, schedules that lack a booster dose tend to elicit weaker protection against colonization [46,58]. Schedules that reduce the number of primary doses could reduce protection against colonization and disease in the first year of life but result in similar responses following receipt of the booster doses [56,59].

The schedules recommended in a country are rarely implemented perfectly. For instance, in a setting with a 3 + 1 recommendation, if children fail to get their booster dose, they will have effectively received a 3 + 0 schedule. This can have important implications for vaccine impact. In the US, communities that had lower uptake of the booster dose had a slower and less-complete elimination of vaccine-targeted serotypes compared with communities with high uptake of the booster [60].

Aside from changes to the pediatric PCV schedule over time, the use of pneumococcal vaccines in adults has also changed. The pneumococcal polysaccharide vaccine (PPSV) has been available for decades. Starting in 2012, PCV13 became available for use in adults, with the US first recommending its use for high-risk groups in 2012 and then for all adults 65+ years of age in 2014. It was recommended to be used in sequence with PCV13 followed by PPSV23 [61].

## Section 2: Hypotheses for why serotype 19F has resurged

There are a number of possible reasons for the resurgence of serotype 19F. The variability between geographic locations with different vaccination schedules where serotype 19F resurged, remained low, or persisted at moderate levels, provides an opportunity to start to understand the patterns. Here, we lay out a number of competing hypotheses, which could also happen concurrently, and arguments for and against each one.

*Hypothesis 1: A new variant of 19F that is more competitive or resistant to vaccine immunity or antibiotics.* If a new variant of 19F is more resistant to antibiotics or immunity from the vaccine or has improved transmissibility, it could result in increased number of 19F cases*.* Serotype 19F is one of the more difficult serotypes to clear with antibodies, as quantified by opsonophagocytic killing assays [62]. Changes in the bacteria that increase the amount of capsule could effectively reduce the effectiveness of the vaccine against colonization [24]. It is also possible that there are unrecognized variants of the polysaccharide structure that render them insensitive to binding of the antibodies [62]. This would be analogous to the situation with serotype 6C, which was not recognized as being distinct from serotype 6A until it was noted that 6C was persisting after vaccine introduction [63]. Finally, changes in antimicrobial susceptibility could give a competitive advantage to certain lineages [64].

*Arguments for:* In Alaska, the majority of serotype 19F isolates identified in nasopharyngeal carriage belonged to a novel lineage (MLST 9074) characterized by a distinct pattern of antibiotic non-susceptibility [16]. In the post-PCV13 era, a multidrug-resistant lineage, GPSC119, has been reported as the predominant cause of 19F disease in England [41], Canada [65], and Australia [38]. Similarly, in South Africa, the GPSC21 lineage has been recognized as a major driver of vaccine-type disease expressing serotype 19F [66].
*Arguments against*: Reported increases in serotype 19F across regions are not attributed to a single lineage (e.g., GPSC119, GPSC1, GPSC21), suggesting that the phenomenon is driven by serotype-specific rather than exclusively genotype-specific factors. Multiple distinct lineages expressing serotype 19F have expanded in the US [67], with varying dominant lineages observed across different countries.
*Additional data that would be helpful*: Long-term sequencing data would allow for evaluation of clonal patterns over time. Experimental data evaluating susceptibility of different lineages to opsonophagocytic killing could help to determine if the dominant lineages now are more resistant to killing, and quantification of polysaccharide production could help to determine if the level of encapsulation is higher.

*Hypothesis 2: Differences in immunogenicity, activity, or duration of protection between PCVs*. PCV7 and PCV13 differ in the strength of immune responses they generate. Compared to PCV7, PCV13 generally produces stronger immunoglobulin G (IgG) responses following receipt of the booster dose, but similar or weaker responses after receipt of the primary series [68]. As a result, communities with inadequate uptake of the booster dose could be more susceptible to increased transmission of serotype 19F among children. Likewise, even if immunogenicity were the same between PCVs, if uptake of the booster declined in a population, it could erode long-term protection against colonization. Serotype 19F requires a high-level of antibodies for protection, so any erosion of immunogenicity or protection might be expected to be evident first for serotype 19F. This problem of insufficient antibodies levels to protect against IPD is similarly reflected by the persistence of another vaccine serotype 3 which has become a major cause of IPD in many countries.

*Arguments for:* The timing of the increase in several locations coincides with the switch from PCV7 to PCV13.
*Arguments against:* While the immune response against many serotypes was lower for PCV13 compared with PCV7, this was not the case for 19F. In a network meta-analysis of head-to-head trials of PCVs, IgG antibody responses against serotype 19F were similar for all PCVs at 28 days post-primary series. However, before and one month after receipt of the booster, the response against 19F was superior for PCV13 and PCV10 compared with PCV7 [22]. Similarly, clinical trials in Israel reported that PCV13 induced higher post-primary and pre-booster geometric mean concentrations against colonization compared with PCV7 [25], as well as higher IgG responses against 19F colonization than PCV7 [26]. Also, the post-booster memory B cell responses do not differ between PCV7 and PCV13 [69]. Finally, many countries that switched to PCV13 such as Israel (transitioned in 2009), and Germany (transitioned in 2009) have not reported a resurgence in 19F [70].
*Additional data that would be helpful*: Cross-sectional colonization studies that track individual-level vaccination status and that enroll both younger and older children would help to determine the importance of the booster dose and the duration of protection against colonization. Additional data on the duration of immunity and protection against colonization for children who receive only primary doses or children who complete the series would also be helpful. Statistical models with spatially resolved IPD or carriage data that link the distribution of 19F to variation in primary and booster coverage could be used to evaluate this hypothesis.

*Hypothesis 3: Change in the adult vaccination strategy* PPSV23 has effectively reduced vaccine-serotype IPD in most high-income countries among older adults [71–74]. The increase in 19F in the US and other countries also coincided with a switch from the use of PPSV23 only in adults to either PCV13 or a sequence of PCV13 followed by PPSV23 [61]. If this new strategy resulted in lower immune responses against serotype 19F, it could have increased susceptibility to 19F IPD cases.

*Arguments for*: The timing of the start of 19F increase, particularly in the United States, coincided with this shift in vaccination policy [61]. It could also be argued that, for those who received PCV13 followed by PPSV23, the 19F polysaccharide component in PPSV23 may potentially cause immune hyporesponsiveness leading to 19F-specific memory B cells depletion among frail adults [75,76].
*Arguments against:* Generally, the immune response against shared serotypes is similar or higher following a sequence of PCV13 and PPSV23 than against PPSV23 alone [77], and that PCV7 or PCV13 combined with PPSV23 is effective against vaccine-serotype IPD [78,79]. This is seen in both antibody concentrations and memory B-cells [79,80]. Additionally, the magnitude of the observed increase suggests that transmission has increased substantially, not just susceptibility to disease, and this is confirmed by the carriage studies from several locations [16,17].

Hypothesis 4: *Reduced competition in the nasopharynx due to the use of higher-valency vaccines*. Modeling studies have predicted that serotype resurgence could occur when moving to vaccines that target more serotypes due to reduced competition in the nasopharynx [34]. It is also possible that specific interactions (e.g., between 19A and 19F) that result from immunological cross-reactivity or metabolic competition, immunologically-independent competition essential in a reciprocal arrangement of 19A and 19F as colonizers could play a role [81]. By reducing the frequency of 19A colonization through the use of PCV13, the vaccine may effectively eliminate one of the closest ecological competitors of 19F.

*Arguments for:* The timing of the resurgence of 19F IPD cases coincided with the switch from PCV7 to PCV13 where the latter includes serotype 19A.
*Arguments against*: There is no definitive clinical evidence supporting cross-protection against serotype 19A disease by PCV7. On the contrary, increases in IPD by 19A have been documented in certain populations with high PCV7 vaccination coverage, suggesting a lack of such cross-protection and suggesting that antibody-mediated competition might be unlikely.
*Additional data that would be helpful*: It is difficult to directly evaluate this general hypothesis. Data on the timing of the increase in 19F by location: if 19F increased in some locations prior to switching from PCV7 to a higher-valency vaccine or if 19F did not increase at all after the switch, this would help to disprove this hypothesis. Specific interactions between 19A and 19F could be evaluated ecologically, for example, by comparing increases in serotype 19F in countries that switched to PCV10 (which does not include 19A) and PCV13 (which does include serotype 19A) or evaluating local frequencies of 19A and 19F to evaluate inverse relationships. Dynamic transmission models that can re-capitulate the resurgence patterns could provide supporting evidence.

Hypothesis 5: *Reduced natural exposure to 19F leading to lower levels of natural boosting*. Pediatric vaccination with PCV reduces transmission of 19F to older kids and adults, but it also reduces exposure and boosting of 19F immunity. This could effectively make older kids and adults more susceptible to transmit 19F than they had been before. For instance, adults >18 years-old who received their childhood PCV7 may have their direct PCV7 protection waned given the estimated mean duration of 8–9 years of PCV7 protection [3]. This age group may likely have become susceptible to vaccine-serotypes once the booster-dose uptake of the current childhood PCV program became suboptimal as is reported by the CDC [82].

*Arguments for:* A study in Malawi reported that antibody concentrations for PCV13 serotypes declined following the administration of a three-dose primary PCV13 series without a booster, followed by a rebound increase beginning three months later, consistent with natural immunologic boosting [58].
*Arguments against:* The amount of time (at least 10 years) between introduction of PCV7 and resurgence of 19F in the US and Australia is likely too long. Moreover, in an Israeli study, there was no evidence to suggest that natural exposure to 19F resulted in sufficient protection against future acquisition by 19F [83]. The timing of the increase in 19F in the US suggests that it is likely related to switching to PCV13, and not an accumulation of susceptibles (honeymoon effect). An increase shortly after a shift to PCV13 may also suggest interactions with serotype 19A.
*Additional data that would be helpful*: To better understand this hypothesis, historical data on seroincidence of pneumococcal exposure among adults would be needed, including estimates of duration of immunity following colonization.

Hypothesis 6: *Post-COVID spike in influenza/respiratory syncytial virus (RSV) activity*. The risk for pneumococcal carriage and disease increases dramatically following infections with influenza or RSV [84–86]. Certain serotypes, including those with low invasiveness like 19F, have a particularly strong association with influenza and RSV activity [87]. Disruption in influenza and RSV dynamics during post-COVID period may have enhanced the activity of pneumococcal serotype 19F.

*Arguments for:* The increase in 19F has been particularly pronounced during the post-COVID period. Moreover, respiratory viruses are likely to enhance susceptibility to serotypes with lower invasiveness such as 19F compared to other serotypes [87,88].
*Arguments against:* The proportion of IPD cases that can be attributed to influenza/RSV disruption is modest, in contrast to the large increases seen in many locations. The increases in the US and Australia also preceded the COVID-19 pandemic, and post-COVID patterns appear to be a continuation of previous trends.
*Additional data that would be helpful*: Time series analyses that evaluate the seasonal timing of increases in disease caused by serotype 19F in relation to the activity of influenza and RSV during the post-COVID period. Longer-term follow-up data may also reveal if 19F increases persist during weaker influenza seasons.

## Section 3: Implications for resurgence, and what to look for going forward

Corresponding to several hypotheses outlined, it is worth noting that there have been broader demographic and clinical changes in the populations over time. Increases in life expectancy, prevalence of comorbidities, and use of immunosuppressive therapies may all influence the epidemiology of pneumococcal disease. Increasing populations of immunocompromised individuals or those with chronic conditions may become more susceptible to IPD, caused by serotypes with traditionally low invasive potential, due to their hyporesponsiveness to vaccines.

Understanding the drivers of resurgence is critical for vaccine policy moving forward as numerous alternative PCVs that use different strategies come into use. For instance, If the reduced immunogenicity of the primary series is the driver, this could point to the importance of ensuring adequate uptake of the booster dose, or prioritizing vaccines that have stronger immunogenicity against key serotypes. If, instead, resurgence is driven by reduced competition from non-vaccine serotypes, this could cause problems for higher-valency vaccines that would further reduce competition in the nasopharynx. If there is some change in the bacteria rendering the strains more resistant to vaccine-induced immunity, then perhaps the formulation of the vaccines needs to be updated.

With PCV10 (Pneumosil), PCV15, PCV20, and PCV21/V116 now available in many locations, high-quality and timely surveillance data are essential to monitor changes in serotype distribution in both colonization and disease. Depending on which of the proposed mechanisms is most important, there could be additional resurgence that occurs, particularly for highly competitive serotypes that are harder to kill with antibodies. The earliest indication of such patterns is likely to come from colonization data. While most colonization studies focus on children <5 years of age, the experience from Alaska suggests that colonization prevalence in older children or adults should also be monitored.

Finally, when studying phenomena such as resurgence or persistence, it is important not to lose sight of the broader impacts of PCVs. If a particular PCV or vaccine strategy results in resurgence of one or more specific serotypes, this needs to be weighed against the overall reduction seen across all serotypes [89]. In the US, despite the resurgence of serotypes 4 and 19F in adults, rates of IPD remain low for all targeted serotypes in children, and the indirect benefit for adults is still large. However, in other parts of the world including the UK and sub-Saharan Africa, the incidence of IPD in adults is now closer to what it was before the introduction of pediatric PCVs. Higher-valency PCVs could potentially still have a net positive benefit compared to existing PCVs even if they do promote some resurgence due to issues associated with their broader serotype coverage. Timely surveillance data, modeling, and analysis will help to weigh these considerations.

## Conclusions

The unexpected resurgence of serotype 19F poses an important challenge for pneumococcal vaccination. Understanding this phenomenon is critical for predicting what might happen when we introduce other new PCVs and whether we should be concerned about potential resurgence of additional serotypes. There are still many key questions that need to be answered to understand this phenomenon. Many of these questions can be addressed through high-quality surveillance and analysis of existing data.

**Key learning points**

- Serotype 19F has unexpectedly resurged despite continued vaccination that targets it, and its resurgence differs between countries
- Serotype 19F is uniquely difficult to protect against and requires higher antibody concentrations than other serotypes for protection against both colonization and invasive disease.
- Ecological competition between serotypes shapes resurgence patterns when vaccines eliminate certain serotypes and free up nasopharyngeal niche which allows other serotypes to expand.
- Multiple non-mutually exclusive mechanisms could explain the resurgence of serotype 19F in the United States and other settings.
- Surveillance covering a broad age range (beyond young children) will be essential for timely detection of emerging vaccine serotypes given the reported substantial resurgence in older age groups.

**Top five papers**

- Gounder PP, Bruden D, Rudolph K, Zulz T, Hurlburt D, Thompson G, et al. Re-emergence of pneumococcal colonization by vaccine serotype 19F in persons aged ≥5 years after 13-valent pneumococcal conjugate vaccine introduction—Alaska, 2008–2013. Vaccine. 2018;36: 691–697. https://doi.org/10.1016/j.vaccine.2017.12.035
- McFarland M, Szasz TP, Zhou JY, Motley K, Sivapalan JS, Isaacson-Schmid M, et al. Colonization with 19F and other pneumococcal conjugate vaccine serotypes in children in St. Louis, Missouri, USA. Vaccine. 2017;35: 4389–4395. https://doi.org/10.1016/j.vaccine.2017.06.047
- Waghela P, Davis R, Campbell M, Datta R, Hislop MS, Vega NJ, et al. Detection of Pneumococcal Carriage in Asymptomatic Healthcare Workers. Open Forum Infect Dis. 2025;12: ofaf008. https://doi.org/10.1093/ofid/ofaf008
- Melin M, Jarva H, Siira L, Meri S, Käyhty H, Väkeväinen M. Streptococcus pneumoniae Capsular Serotype 19F Is More Resistant to C3 Deposition and Less Sensitive to Opsonophagocytosis than Serotype 6B. Infection and Immunity. 2009;77: 676–684. https://doi.org/10.1128/iai.01186-08
- Dagan R, Patterson S, Juergens C, Greenberg D, Givon-Lavi N, Porat N, et al. Comparative Immunogenicity and Efficacy of 13-Valent and 7-Valent Pneumococcal Conjugate Vaccines in Reducing Nasopharyngeal Colonization: A Randomized Double-Blind Trial. Clin Infect Dis. 2013;57: 952–962. https://doi.org/10.1093/cid/cit428
